# Genome Sequences of Four *Acinetobacter baumannii*-*A. calcoaceticus* Complex Isolates from Combat-Related Infections Sustained in the Middle East

**DOI:** 10.1128/genomeA.00026-14

**Published:** 2014-02-06

**Authors:** Patrick Ketter, M. Neal Guentzel, James P. Chambers, James Jorgensen, Clinton K. Murray, Andrew P. Cap, Jieh-Juen Yu, Mark Eppinger, Bernard P. Arulanandam

**Affiliations:** a University of Texas at San Antonio, San Antonio, Texas, USA; b University of Texas Health Science Center at San Antonio, San Antonio, Texas, USA; c San Antonio Military Medical Center, JBSA-Fort Sam Houston, San Antonio, Texas, USA; d United States Army Institute of Surgical Research, San Antonio Military Medical Center, JBSA-Fort Sam Houston, San Antonio, Texas, USA

## Abstract

*Acinetobacter baumannii* is among the most prevalent bacterial causes of combat-related infections on the battlefield. Antibiotic resistance and a poor understanding of the protective host immune responses make treatment difficult. Here, we report the genome sequences of four clinical *Acinetobacter baumannii-A. calcoaceticus* complex isolates exhibiting significant differences in virulence in a mouse sepsis model.

## GENOME ANNOUNCEMENT

*Acinetobacter baumannii* accounts for >36% of combat-related infections resulting from injuries sustained by military service personnel in the Middle East, making it among the most prevalent bacterial pathogens encountered on the battlefield (1, 2). Ubiquitous in nature, *A. baumannii* resists desiccation, allowing the pathogen to survive in relatively arid environments most other bacteria cannot (3–5). This, in combination with the increased level of antibiotic resistance associated with *A. baumannii* and its ability to colonize various bodily sites, makes these infections difficult to control and treat (3, 6–11). The limited genomic plasticity and biochemical conservation among *Acinetobacter* species pose problems with regard to species identification in clinical settings, resulting in *A. baumannii* and *Acinetobacter calcoaceticus* often being reported as the *A. baumannii-A. calcoaceticus* complex (ABC) (10, 12). Genomic heterogeneity in *A. baumannii* has an established impact on patient outcome based on variations of the virulence and antibiotic resistance gene alleles present in each strain (13–16).

To this end, we sequenced four clinical multidrug-resistant ABC strains, CI77, CI78, CI79, and CI86, from a collection obtained from respiratory (CI78 and CI79) or wound (CI77 and CI86) cultures from military personnel injured in either Iraq or Afghanistan (17). In a murine sepsis model, we observed significant differences in virulence *in vivo* between strains CI77 and CI79 correlating to sustained and elevated blood serum levels of an acute-phase small pattern recognition receptor known as PTX3; PTX3 possesses antimicrobial properties following challenge with strain CI79 (P. M. Ketter, M. N. Guentzel, B. Schaffer, M. Herzig, X. Wu, C. G. Fedyk, J. Yu, J. Jorgensen, J. P. Chambers, A. P. Cap, and B. P. Arulanandam, submitted for publication). Genomic DNA was subjected to Illumina sequencing using paired-end libraries with 300-bp inserts on the HiSeq 2000 platform. The draft genome was assembled with the Velvet assembler (18, 19), and the IGS Annotation Engine and Manatee were used for structural and functional genome annotation and visualization of the chromosomes and plasmid contigs (20).

The observed genome sizes of the isolates range from 3.8 to 4.2 Mb, with an average G+C content of 38%. *In silico* comparison of the 16s rRNA genes allowed the complex and species attribution of CI77, CI79, and CI86 as being pathogenic *A. baumannii* strains, while CI78 is representative of *A. calcoaceticus*, is generally considered nonpathogenic, and is found to occasionally colonize but rarely cause infection in humans (10, 12). We determined the carriage of several reported *A. baumannii* virulence factors, such as LpxC, PglL, and AbaI (21–23), and found only limited genomic plasticity among the analyzed Middle Eastern ABC isolates. The availability of these sequences as a reference in RNAseq analysis will assist in future studies examining alterations in the ABC complex transcriptome and will help enhance our understanding of the underlying genetics and regulatory pathways leading to altered clinical disease manifestation in patients infected with emerging pathogens of ABC.

### Nucleotide sequence accession numbers.

The genome sequences of the *Acinetobacter* clinical isolates CI77, CI78, CI79, and CI86 have been deposited in GenBank under accession no. AVOC00000000, AVOE00000000, AVOD00000000, and AVOB00000000, respectively.
